# 2904. Use Of Biofire® Joint Infection Filmarray for the Microbiological Diagnosis of Acute Hematogenous Septic Arthritis in Children

**DOI:** 10.1093/ofid/ofad500.175

**Published:** 2023-11-27

**Authors:** Alison Boast, Gena Gonis, Melisa Gauci, Sunitha Velagapudi, Peter C Harris, Michael B Johnson, Nigel Curtis, Andrew J Daley, Amanda Gwee

**Affiliations:** Royal Children's Hospital Melbourne, Melbourne, Victoria, Australia; Royal Children's Hospital Melbourne, Melbourne, Victoria, Australia; Royal Children's Hospital Melbourne, Melbourne, Victoria, Australia; Royal Children's Hospital Melbourne, Melbourne, Victoria, Australia; Royal Children's Hospital Melbourne, Melbourne, Victoria, Australia; Royal Children's Hospital Melbourne, Melbourne, Victoria, Australia; Royal Children's Hospital Melbourne, Melbourne, Victoria, Australia; The Royal Children's Hospital, Melbourne, Victoria, Parkville, Victoria, Australia

## Abstract

**Background:**

Making a microbiological diagnosis in paediatric joint infections is challenging due to the high proportion of fastidious pathogens, in particular *Kingella kingae*. The Biofire® Joint Infection (JI) Panel allows the rapid molecular diagnosis of a wide spectrum of organisms implicated in pediatric acute haematogenous septic arthritis.

**Methods:**

This study was done in children enrolled in the BEST trial (NCT04538053), a non-inferiority trial comparing entirely oral to intravenous antibiotic treatment of acute bone and joint infections. When sufficient synovial fluid was available after routine tests, the Biofire JI panel was done in parallel with culture. As the assay was used prior to Therapeutic Goods Administration registration as an *in vitro* diagnostic device, it was not released to clinicians.

**Results:**

Overall, 16 joint fluid samples from 16 children with a median age of 2.3 (range 1 to 13.3) years were included. All children had pleocytosis on microscopy. Standard bacterial culture and Biofire JI panel had concordant results in nine children – *K. kingae* (3), *Streptococcus pyogenes* (1), *Staphylococcus aureus* (1), and negative (4). In six samples, a pathogen was identified by Biofire when culture was negative (4 *K. kingae*, 1 *Streptococcus pneumoniae*, 1 *S. aureus*). One child cultured *S. aureus* that was not detected by Biofire.

**Conclusion:**

Biofire JI panel improved the microbiological diagnostic yield from synovial fluid samples from children with septic arthritis. Larger studies are required to determine the influence on clinical practice.

**Disclosures:**

**All Authors**: No reported disclosures

